# Impairment of quality of life due to COVID-19-induced long-term olfactory dysfunction

**DOI:** 10.3389/fpsyg.2023.1165911

**Published:** 2023-04-20

**Authors:** Anja L. Winter, Sofie Henecke, Johan N. Lundström, Evelina Thunell

**Affiliations:** ^1^Department of Clinical Neuroscience, Karolinska Institutet, Stockholm, Sweden; ^2^Monell Chemical Senses Center, Philadelphia, PA, United States; ^3^Stockholm University Brain Imaging Centre, Stockholm University, Stockholm, Sweden; ^4^Department of Psychological Sciences, Purdue University, West Lafayette, IN, United States

**Keywords:** olfactory disorders, parosmia, phantosmia, quality of life, COVID-19

## Abstract

**Introduction:**

Olfactory dysfunction is one of many long-lasting symptoms associated with COVID-19, estimated to affect approximately 60% of individuals and often lasting several months after infection. The associated daily life problems can cause a decreased quality of life.

**Methods:**

Here, we assessed the association between perceived quality of life and both qualitative and quantitative olfactory function (distorted and weakened sense of smell, respectively) in 58 individuals who had undergone confirmed SARS-CoV-2 infection and who complained about olfactory dysfunction.

**Results:**

Participants with large quantitative olfactory dysfunction experienced a greater reduction in their quality of life. Moreover, our participants had a high prevalence of qualitative olfactory dysfunction (81%) with a significant correlation between qualitative olfactory dysfunction and daily life impairment. Strong drivers of low quality of life assessments were lack of enjoyment of food as well as worries related to coping with long-term dysfunctions.

**Discussion:**

These results stress the clinical importance of assessing qualitative olfactory dysfunction and the need to develop relevant interventions. Given the poor self-rated quality of life observed, healthcare systems should consider developing support structures, dietary advice, and guidelines adapted to individuals experiencing qualitative olfactory dysfunction.

## Introduction

The COVID-19 pandemic has raised public awareness of olfaction and its importance for our health, wellbeing, and quality of life (Elkholi et al., 2021). One common acute symptom related to COVID-19 is olfactory dysfunction (Lechien et al., 2020), estimated to affect up to 70% of individuals with mild to moderate symptoms (Vaira et al., 2020). Many recover after a few days, but recent follow-up studies show that some patients still experience olfactory dysfunction 2 years after infection (McWilliams et al., 2022).

The mechanisms behind the pathophysiology of long-lasting olfactory dysfunction related to COVID-19 is still not known. However, reports of specific brain changes following infection have been observed. For example, COVID-19 patients with olfactory dysfunction display reductions in functional connectivity between the orbitofrontal cortex and dorsal anterior cingulate cortex (Wingrove et al., 2023) as well as decreased gray matter volume surrounding olfactory-related regions such as the orbitofrontal cortex and parahippocampal gyrus (e.g., Douaud et al., 2022; Campabadal et al., 2023). These patients also show reduced blood flow in the orbital and medial frontal regions (Yus et al., 2022). In line with the notion that central dysfunction is the cause of long-term olfactory loss is data showing that when comparing pre- and post COVID-19 changes, the olfactory bulb volume is reduced in nearly all cases (Thunell et al., 2022).

Although central causes are reported in the literature, multiple causes linked to abnormalities in the peripheral system have also been reported (e.g., Finlay et al., 2022; Zazhytska et al., 2022) and it is likely that both peripheral and central mechanisms are at play.

The sense of smell provides important information about our environment and guides attention via perceived valence of odor sources, which allows us to avoid threats and approach rewards (Croy et al., 2014). For instance, olfaction plays a crucial role in assessing the edibility of an item (Stevenson, 2010) and is also protective by alerting to hazards, such as fire or gas (Pence et al., 2014). Olfactory dysfunction therefore incurs an increased risk of exposure to environmental hazards as well as food poisoning (Pence et al., 2014). Moreover, olfactory dysfunction is linked to impairments in both daily functioning and interpersonal relationships (Erskine and Philpott, 2020), which may negatively affect both physical and psychological health (Elkholi et al., 2021). Accordingly, people with long-term smell loss often exhibit depressive symptoms, diminished self-esteem, loss of intensity of emotional experiences (Schäfer et al., 2021), and lower overall quality of life (Miwa et al., 2001; Croy et al., 2014).

Most studies on olfactory problems focus on quantitative dysfunction, i.e., hyposmia (decreased sensitivity) and anosmia, so-called “smell blindness.” However, COVID-19 has been reported to also cause qualitative olfactory dysfunction, i.e., parosmia (distorted smells) and phantosmia (odor hallucinations) in around 40–50% of individuals who experience decreased sensitivity during or after the infection (Hopkins et al., 2021; Frasnelli et al., 2022). Qualitative olfactory impairments often onset months after infection, may last for a long time (Gary et al., 2022), and have been reported to have a stronger negative impact on the quality of the individual’s life than quantitative dysfunctions alone (Leopold, 2002; Frasnelli and Hummel, 2005). Indeed, COVID-19 patients with parosmia show reduced quality of life and rate their situation as worse than do those without parosmia (Otte et al., 2022).

COVID-19-related reductions in quality of life are well described in the literature, as are the negative effects of an impaired sense of smell on quality of life, but it is still unclear which specific aspects of COVID-19 related olfactory dysfunction are related to prolonged decreased quality of life. Here, we assessed qualitative and quantitative olfactory dysfunction in individuals who had previously undergone SARS-CoV-2 infection and hypothesized a positive correlation between the former and daily life impairment. Identifying the causes of decreased quality of life will aid risk prediction and facilitate the development of interventions.

## Methods

### Participants

Participants (*n* = 138) were recruited from the longitudinal COMMUNITY (COVID-19 Immunity) Study, in which all participants continuously have been tested for seroprevalence of SARS-CoV-2 antibodies since the beginning of the pandemic (Rudberg et al., 2020). Two of these were excluded due to problematic testing conditions and one due to being diagnosed with a disorder known to change the sense of smell. None of the individuals suffered from nasal congestion or rhinorrhoea, conditions associated with olfactory dysfunction (Landis et al., 2003; Doty and Kamath, 2014). Another 40 participants had never tested positive for SARS-CoV-2 antibodies and were therefore excluded. From the remaining 95 participants who had at some point tested positive for SARS-CoV-2 antibodies, only participants who experienced smell/taste-related problems (58) were instructed to fill out the form related to daily life impairment (QOD-NS; Table 1). The final dataset used in this study thus consists of 58 individuals. Detailed information related to the time since onset of COVID-19 was missing for 8 out of these participants. The study was approved by the Swedish Ethical Review Authority (Dnr: 2021-02052) and all participants provided written informed consent prior to participation. All procedures were in accordance with the Helsinki declaration. See Table 1 for details related to the participants.

**Table 1:** Descriptive statistics of research participants.

	Both	Qualitative	Quantitative	None	Total
	(*N* = 19)	(*N* = 28)	(*N* = 4)	(*N* = 7)	(*N* = 58)
Sex					
Female	16 (84.2%)	24 (85.7%)	4 (100%)	6 (85.7%)	50 (86.2%)
Male	3 (15.8%)	4 (14.3%)	0 (0%)	1 (14.3%)	8 (13.8%)
Age (years)					
Mean (SD)	48.5 (11.7)	47.8 (11.2)	54.0 (11.0)	46.4 (11.0)	48.3 (11.2)
Time since COVID-19 (days)					
Mean (SD)	458 (30.1)	441 (68.3)	448 (45.3)	501 (34.0)	456 (54.6)

The group label Both indicates participants who were classified with both qualitative and quantitative olfactory dysfunction, and the group label None indicates participants who were classified with neither. The label Qualitative indicates participants with only qualitative dysfunction, and the label Quantitative indicates participants with only quantitative dysfunction.

### Measurement

#### Qualitative olfactory dysfunction

To identify participants with qualitative olfactory dysfunctions, we used a questionnaire containing two dichotomous questions; (1) “Do you experience olfactory distortions, i.e., that smells have changed after COVID” and (2) “Do you experience phantosmia after COVID (olfactory hallucinations/phantom smells)?” An affirmative answer to question 1 categorized the participants as parosmic and an affirmative answer to question 2 categorized them as phantosmic. The participants additionally answered four structured questions about their experienced degree of qualitative olfactory dysfunction (Landis et al., 2010) each with four response alternatives; this is never the case (assigned 1 point); this is rarely the case (2 points), this is often the case (3 points), this is always the case (4 points), yielding a minimum qualitative olfactory dysfunction score of 4 and a maximum of 16. Note that this scale is reversed as compared to Landis et al. (2010).

#### Quantitative olfactory dysfunction

We assessed quantitative olfactory ability using the Sniffin’ Sticks extended test battery (Burghart Messtechnik, Holm, Germany), a validated psychophysical measure of olfactory ability (Hummel et al., 1997; Kobal et al., 2000; Sorokowska et al., 2015) commonly used to quantify olfactory deficits in COVID-19 patients (e.g., Iannuzzi et al., 2021; Prem et al., 2021; Stankevice et al., 2023). The test consists of a nasal chemosensory performance assessment utilizing felt tip pen-like devices for odor presentation and includes three subtests measuring odor threshold (T), odor discrimination (D), and odor identification (I); yielding a summarized (TDI) score of olfactory function where higher scores indicate better function. In the present study, the session begun with an odor threshold subtest using 16 triplets of pens where one pen in each triplet contained n-butanol and two were odorless. The task of the participant was to identify the pen with the odor when an experimenter presented consecutive triplets in a staircase procedure. The second subtest was focused on odor discrimination and contained 16 triplets of pens with various odorants. Two pens in each triplet contained the same odorant and the participant was instructed to select the pen that smelled different. The final subtest, an odor identification task, included 16 pens with everyday odors. Participants were instructed to identify the odors using a multiple-choice answering format with a four-alternative card for each odor. All three subtests employed a forced-choice answering format. Based on normative data (Oleszkiewicz et al., 2019), anosmia was defined as a TDI score of ≤16, normosmia as a score of ≥30.75, and hyposmia as a score between these two values. Total testing time for each subject was approximately 1 h.

#### Daily life impairment

Self-assessment of daily life impairment related to olfactory dysfunction was performed using a Swedish translation of the shorter modified (Simopoulos et al., 2012) Questionnaire of Olfactory Disorders – Negative Statements subscale (QOD-NS) (Frasnelli and Hummel, 2005), a widely used questionnaire evaluating the negative impact of smell loss on quality of life. The measure is a four-scale questionnaire targeting the degree of experienced suffering related to olfactory dysfunction by utilizing a Likert-scale based on 17 items where participants could either agree (3 points), partly agree (2 points), partly disagree (1 point), or disagree (0 points) with various statements. The final score varies from a minimum of 0 and a maximum of 51, with higher scores indicating more severe daily life impairment.

### Statistical analyses

All data and analyses included in this manuscript can be accessed from the Open Science Framework (OSF) at https://osf.io/czeq3/?view_only=8ad63cac2cd94121b954f47a403fab0e. Statistical analyses were performed using the statistical software R (v4.2.2; R Core Team, 2022) and the packages cocor (v1.1.4; Diedenhofen and Musch, 2015), dplyr (v1.0.10; Wickham et al., 2022a), ggplot2 (v3.4.0; Wickham, 2016), ggridges (v0.5.4; Wilke, 2022), haven (v2.5.1; Wickham et al., 2022b), likert (v1.3.5; Bryer and Speerschneider, 2016), psych (v2.2.9; Revelle, 2022), table1 (v1.4.2; Rich, 2021), and tidyr (v1.2.1; Wickham and Girlich, 2022). Calculation for the test of the difference between two dependent correlations with one variable in common was carried out using quantpsy.org computer software (Lee and Preacher, 2013). The significance criterion for all statistical tests was set to *α* = 0.05.

## Results

### Qualitative olfactory dysfunction

We first set out to determine the prevalence of qualitative olfactory dysfunction (parosmia; distorted odor perception and phantosmia; phantom smells) in our sample based on participants’ subjective answers to the questionnaire. Forty-seven out of 58 individuals (81%) experienced qualitative problems, out of which 21 individuals reported both parosmia and phantosmia, 25 only parosmia, and one only phantosmia. Further, there was a large co-occurrence of quantitative and qualitative olfactory dysfunction (Table 2). Seven of the participants included in this analysis were classified as having neither quantitative nor qualitative dysfunction, despite reporting that they experienced problems.

**Table 2:** Daily life impairment (QOD-NS), quantitative (TDI) and qualitative (olfactory dysfunction score) olfactory measures grouped by olfactory dysfunction.

	Both (*N* = 19)	Qualitative (*N* = 28)	Quantitative (*N* = 4)	None (*N* = 7)	Total (*N* = 58)
QOD-NS					
Mean (SD)	16.8 (9.97)	11.0 (7.95)	4.50 (5.26)	6.57 (4.24)	11.9 (8.97)
TDI					
Mean (SD)	23.1 (5.99)	33.9 (2.37)	28.8 (1.02)	33.9 (3.21)	30.0 (6.36)
Qualitative olfactory dysfunction score					
Mean (SD)	9.42 (2.32)	8.36 (2.50)	4.25 (0.500)	5.43 (1.27)	8.07 (2.71)

The group label Both indicates participants who were classified with both qualitative and quantitative olfactory dysfunction, and the group label None indicates participants who were classified with neither. The label Qualitative indicates participants with only qualitative dysfunction, and the label Quantitative indicates participants with only quantitative dysfunction.

### Quantitative olfactory dysfunction

Next, we assessed quantitative olfactory dysfunction, as defined by the TDI scores. Twenty-three (40%) of our participants scored in accordance with quantitative olfactory dysfunction; 20 were classified as hyposmic (weakened sense of smell) and 3 were classified as anosmic (unable to use their sense of smell). Overall, TDI scores ranged from 12 to 40. See Table 2 for details.

### Daily life impairment

**Figure 1:**
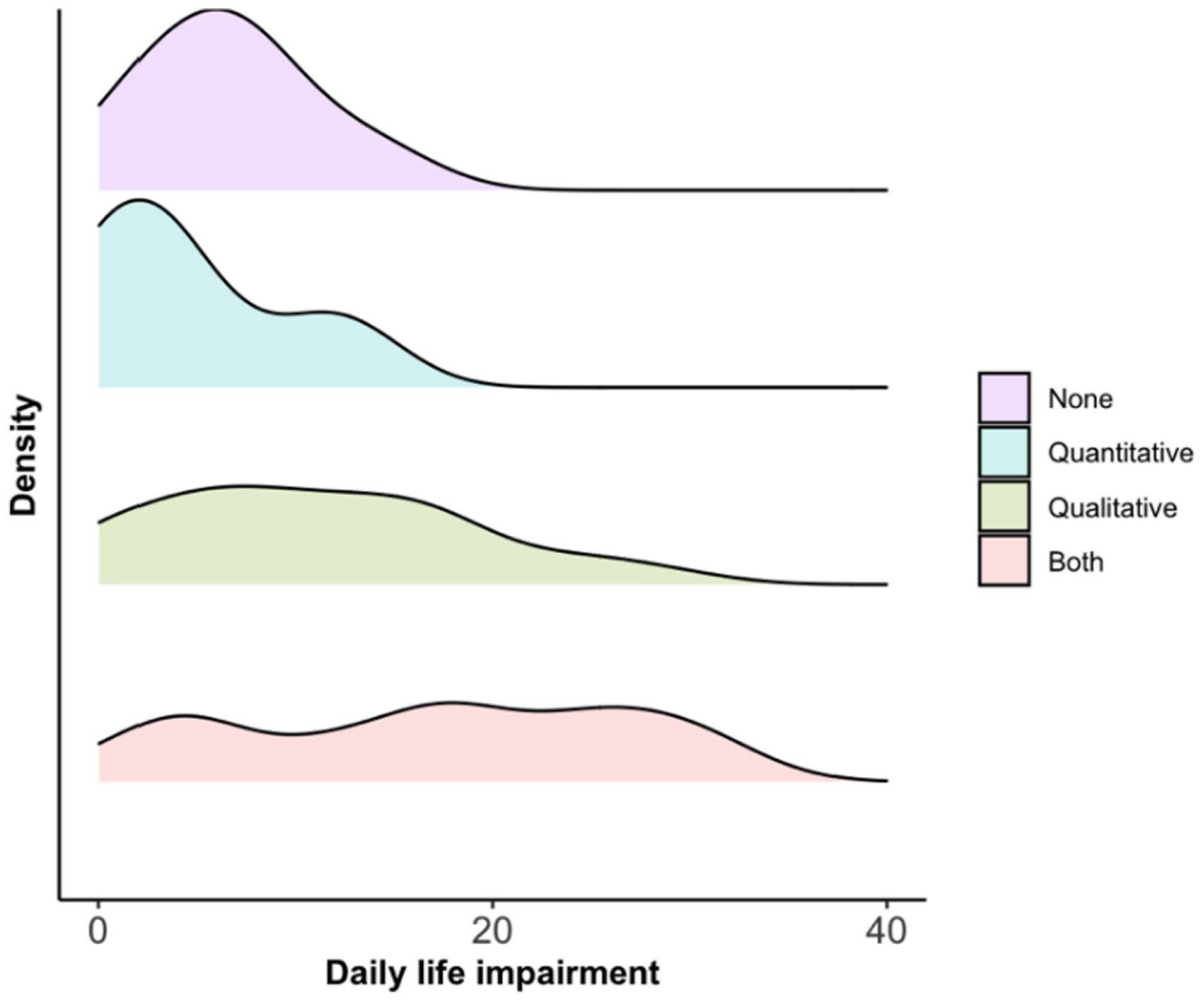
Density of distribution of average daily life impairment (QOD-NS score) assessments per olfactory dysfunction group.

Last, we computed quality of life impairment scores based on the QOD-NS questionnaires to assess how it is influenced by the qualitative and quantitative olfactory impairments (Table 2). As can be seen in Figure 1, the distributions of QOD-NS scores differed between clinical groups with a wider tail distribution and more extreme values for participants with qualitative and those with both qualitative and quantitative problems as compared with participants with quantitative or no impairment.

**Figure 2:**
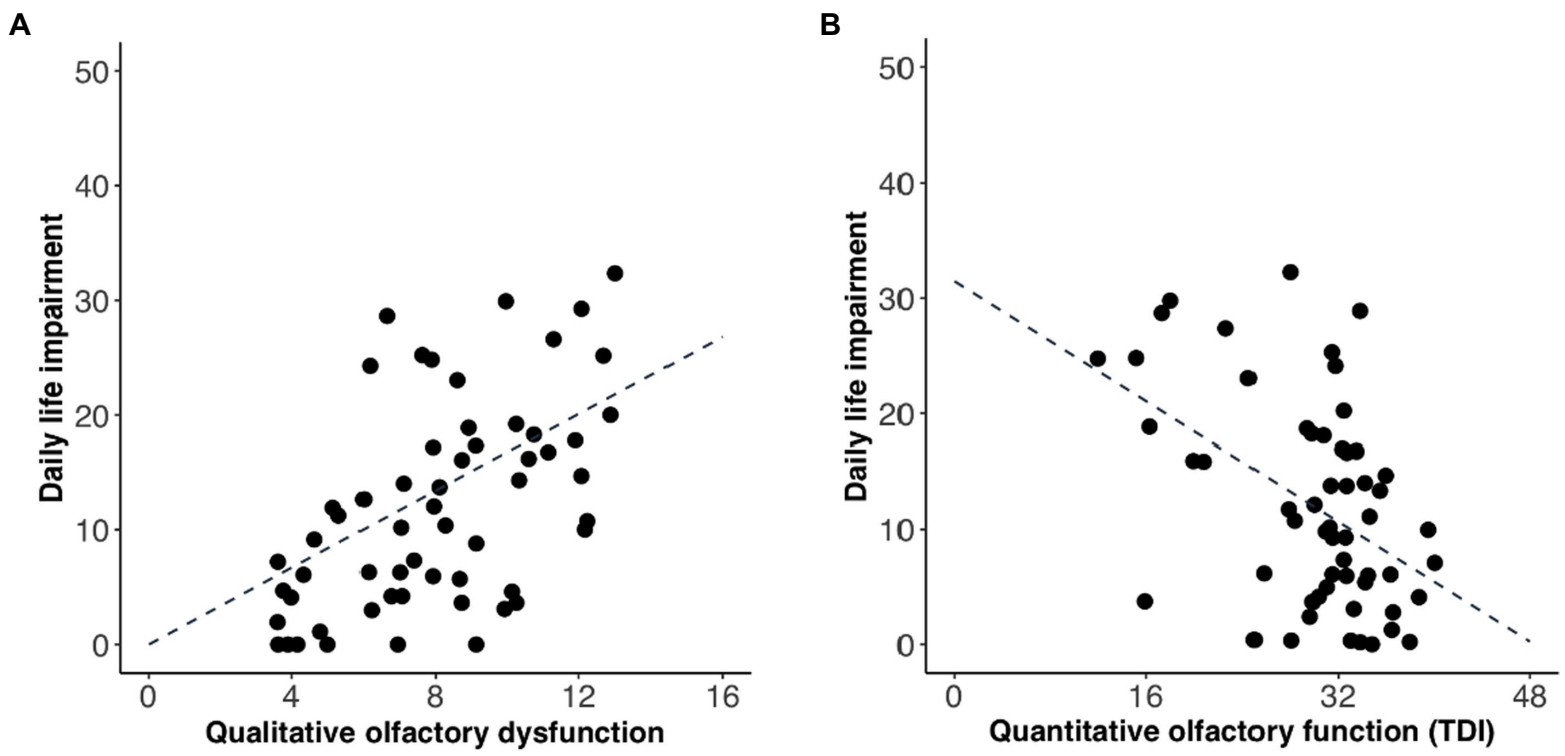
Relationship between average daily life impairment (QOD-NS score) and **(A)** degree of qualitative olfactory dysfunction and **(B)** quantitative olfactory function. Each data point represents one participant. Regression line is indicated by the intersected line. Scores are slightly jittered for visibility.

Next, we wanted to know whether there was a link between degree of impairment and the individuals’ rated quality of life. Using Spearman’s rank correlation, we found that daily life impairment was positively correlated with the degree of qualitative olfactory dysfunction (*r* = 0.57, *p* < 0.001; Figure 2A). Similarly, a correlation was found between daily life impairment and quantitative olfactory function (*r* = −0.38, *p* < 0.005; Figure 2B).

**Figure 3:**
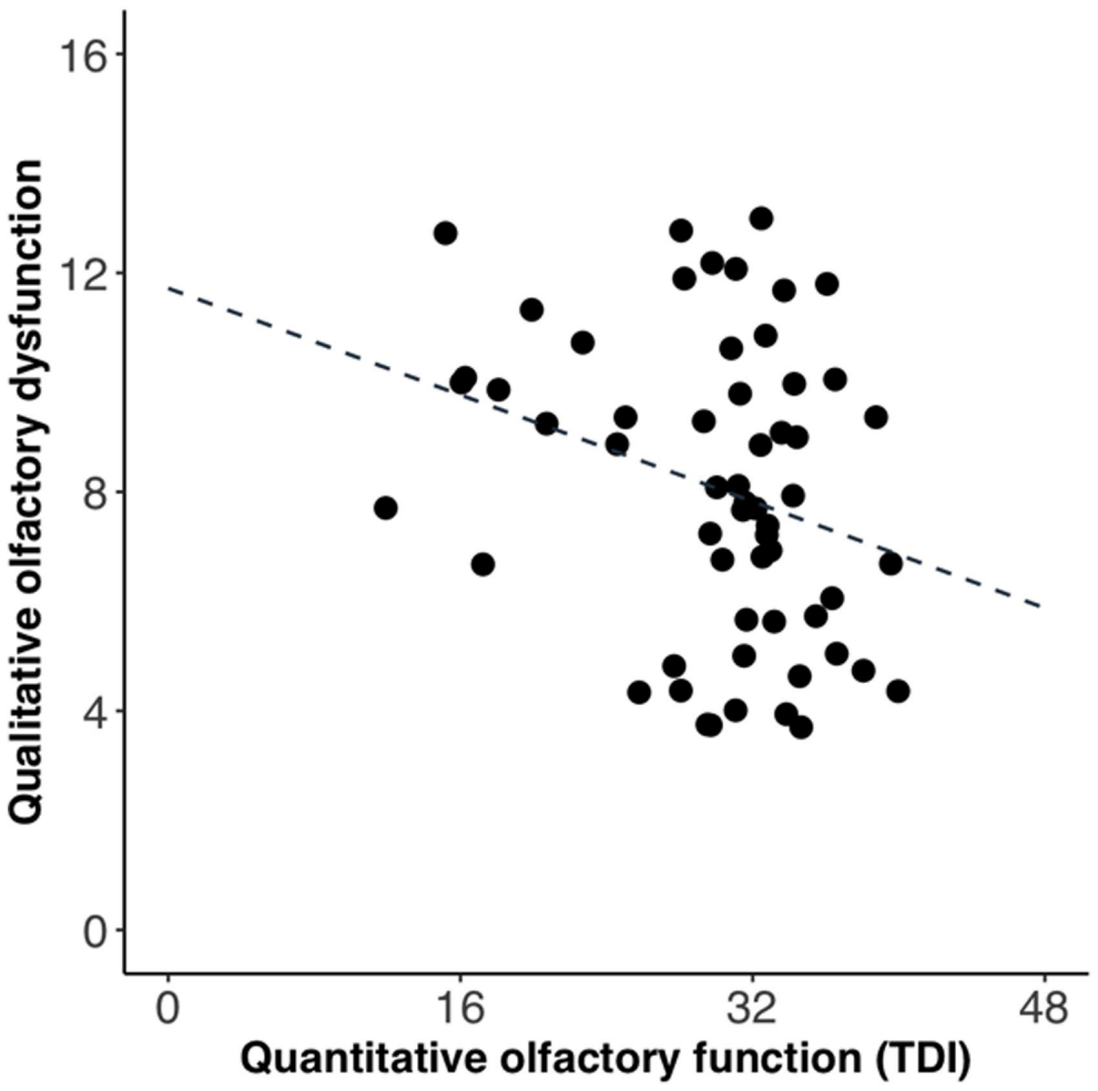
Relationship between qualitative olfactory dysfunction and quantitative olfactory function. Each data point represents one participant. Regression line is indicated by the intersected line. Scores are slightly jittered for visibility.

To compare if daily life impairment had a significantly larger association with qualitative than quantitative olfactory dysfunction, we carried out a Fisher’s r-to-z transformation followed by Steiger’s (1980) equations to compute asymptotic covariance of the estimates. The difference between the correlation coefficients linking daily life impairment to qualitative and quantitative dysfunction, respectively, was not significant (*z* = 1.41, *p* = 0.16). In our sample, the qualitative olfactory dysfunction and quantitative olfactory function were correlated (*r* = −0.26, *p* < 0.05; Figure 3), meaning that there was some degree of comorbidity which might make separate assessments problematic.

**Figure 4:**
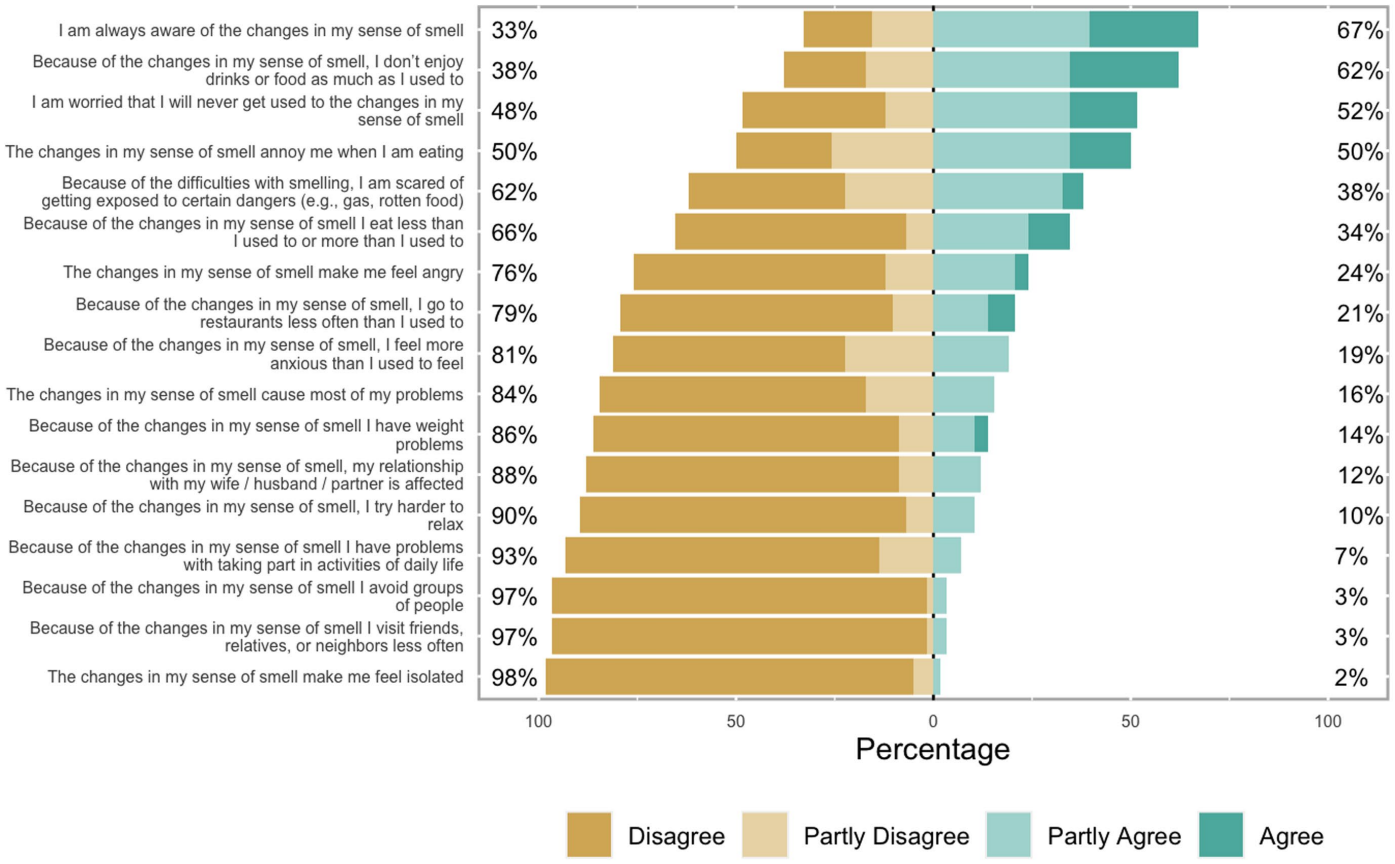
Response frequency to the questions of QOD-NS.

To better understand what aspects of daily life were impaired, we also looked for trends in the answers to the specific questions of the QOD-NS. We found that negative experiences related to eating seemed like the most prevalent theme, whereas problems concerning relationships or changes in social behavior were rare (Figure 4).

## Discussion

Here we show that 80% of individuals with lingering olfactory dysfunction from a COVID-19 infection still experience associated impairments in their quality of life more than a year after infection. To a great extent, this is due to their qualitative olfactory dysfunction. Qualitative olfactory dysfunction can be a debilitating condition, previously shown to correlate with higher rates of anxiety and depression (Philpott and Boak, 2014). In our sample, qualitative olfactory dysfunction was twice as common as quantitative dysfunction, and only four participants suffered from quantitative olfactory dysfunction without experiencing also qualitative olfactory dysfunction. Our data confirm that the severity of this prevalent qualitative olfactory dysfunction is positively correlated with daily life impairment. This is explained by specific themes related to daily life impairment, where daily life seems to be most negatively impacted by a change in eating patterns; potentially because social situations involving eating tend to be more affected by qualitative changes in smell than quantitative problems. For example, many individuals with parosmia are unable to ingest certain food items because they are disgusted by the smell, whereas hyposmia will not elicit the same strong affective reaction. Although daily life impairment seemed more strongly associated with qualitative dysfunction than quantitative dysfunction, no significant difference was found between the correlations. Therefore, we cannot conclude that daily life impairment is associated with qualitative dysfunction to a greater extent than with quantitative dysfunction. However, it is worth noting that there was a considerable comorbidity between the two diagnoses meaning that a firm separation is difficult to achieve.

Considerable similarities between COVID-19-associated olfactory dysfunction and other types of post-viral olfactory dysfunction have previously been established via meta-analysis (Imam et al., 2020). There is therefore no reason to believe that our results are limited to COVID-19-related olfactory dysfunction, but rather they likely apply also to smell-related problems caused by other viral infections. However, olfactory dysfunctions due to other reasons such as head trauma or neurodegenerative disorders may yield other results. In our sample, it appears that those with no olfactory dysfunction reported higher average daily life impairment scores than the group with quantitative dysfunction. One reason why these normosmic individuals experienced a decreased quality of life may be that they noticed a decrease in olfactory function compared to their pre-COVID-19 olfactory function. However, firm conclusions based on this small sample size should be avoided.

A recent meta-analysis suggested that women are less likely than men to regain their sense of smell (Tan et al., 2022), which might partially explain the large proportion of women signing up for the current study. However, the uneven sex distribution might also simply be due to the skewed sex balance of the population of healthcare workers from which the sample was taken. The strength of this study is the extensive psychophysical testing done in a homogenous group that was continuously monitored for COVID-19 infection from the onset of the pandemic. As mentioned previously, disruptions of daily life related to qualitative olfactory dysfunction may cause mental health related problems (e.g., Miwa et al., 2001; Croy et al., 2014; Elkholi et al., 2021; Schäfer et al., 2021). Recent data show that individuals experiencing olfactory dysfunction also report a lack of support from the medical field (Ball et al., 2021; Kye Wen Tan et al., 2022), providing incentive to further investigate the condition and develop evidence-based treatment specifically targeting qualitative olfactory dysfunction. Moreover, the present study did not exclude, nor control for, participants with long-covid syndrome or other related symptoms. Recent studies have shown associations between olfactory-related quality of life and affective as well as cognitive dysfunctions. For example, COVID-19 related olfactory dysfunction has been related to mood disturbances (Llana et al., 2023), a higher likelihood of depression (Liu et al., 2022), as well as cognitive dysfunction (Delgado-Alonso et al., 2022). The observed relationship between olfactory dysfunction and quality of life could therefore be mediated by other affective or cognitive symptoms. Hopefully, future studies will be able to replicate this type of extensive testing on highly controlled groups in a larger sample.

In conclusion, COVID-19 can cause long-lasting problems, and a large number of recovering individuals still experience olfactory dysfunction more than a year after infection. We found that individuals who suffer from lingering qualitative olfactory dysfunction experience limitations in daily life, in particular related to food and eating. Because qualitative olfactory dysfunction is known to be associated also with depression and anxiety, our results further stress the clinical importance of acknowledging it for risk predictions in future clinical research; as well as in the development of new interventions, such as support structures, dietary advice, and guidelines.

## Data availability statement

The datasets presented in this study can be found in online repositories. The names of the repository/repositories and accession number(s) can be found at: https://osf.io/czeq3/?view_only=8ad63cac2cd94121b954f47a403fab0e.

## Ethics statement

The studies involving human participants were reviewed and approved by the Swedish Ethical Review Authority (Dnr: 2021-02052). The patients/participants provided their written informed consent to participate in this study.

## Author contributions

JL contributed to conception and design of the study. AW collected the data, performed the statistical analysis, and wrote the first draft of the manuscript. ET, JL, and SH wrote sections of the manuscript. All authors contributed to manuscript revision, read, and approved the submitted version.

## Funding

Funding provided by grants awarded to JL from the Knut and Alice Wallenberg Foundation (KAW 2018.0152), the Swedish Research Council (2021-06527), and a donation from Stiftelsen Bygg-Göta för Vetenskaplig forskning.

## Conflict of interest

The authors declare that the research was conducted in the absence of any commercial or financial relationships that could be construed as a potential conflict of interest.

## Publisher’s note

All claims expressed in this article are solely those of the authors and do not necessarily represent those of their affiliated organizations, or those of the publisher, the editors and the reviewers. Any product that may be evaluated in this article, or claim that may be made by its manufacturer, is not guaranteed or endorsed by the publisher.
